# HSP90 inhibition sensitizes head and neck cancer to platin-based chemoradiotherapy by modulation of the DNA damage response resulting in chromosomal fragmentation

**DOI:** 10.1186/s12885-017-3084-0

**Published:** 2017-01-31

**Authors:** Martin McLaughlin, Holly E. Barker, Aadil A. Khan, Malin Pedersen, Magnus Dillon, David C. Mansfield, Radhika Patel, Joan N. Kyula, Shreerang A. Bhide, Kate L. Newbold, Christopher M. Nutting, Kevin J. Harrington

**Affiliations:** 1 Targeted Therapy Team, The Institute of Cancer Research, Chester Beatty Laboratories, 237 Fulham Road, London, SW3 6JB UK; 2 The Royal Marsden Hospital, 203 Fulham Road, London, SW3 6JJ UK; 3 Division of Radiotherapy and Imaging, The Institute of Cancer Research, 237 Fulham Road, London, UK

**Keywords:** RAD51, FANCA, ATM, AUY922, HNSCC, DDR

## Abstract

**Background:**

Concurrent cisplatin radiotherapy (CCRT) is a current standard-of-care for locally advanced head and neck squamous cell carcinoma (HNSCC). However, CCRT is frequently ineffective in patients with advanced disease. It has previously been shown that HSP90 inhibitors act as radiosensitizers, but these studies have not focused on CCRT in HNSCC. Here, we evaluated the HSP90 inhibitor, AUY922, combined with CCRT.

**Methods:**

The ability of AUY922 to sensitize to CCRT was assessed in p53 mutant head and neck cell lines by clonogenic assay. Modulation of the CCRT induced DNA damage response (DDR) by AUY922 was characterized by confocal image analysis of RAD51, BRCA1, 53BP1, ATM and mutant p53 signaling. The role of FANCA depletion by AUY922 was examined using shRNA. Cell cycle checkpoint abrogation and chromosomal fragmentation was assessed by western blot, FACS and confocal. The role of ATM was also assessed by shRNA. AUY922 in combination with CCRT was assessed in vivo.

**Results:**

The combination of AUY922 with cisplatin, radiation and CCRT was found to be synergistic in p53 mutant HNSCC. AUY922 leads to significant alterations to the DDR induced by CCRT. This comprises inhibition of homologous recombination through decreased RAD51 and pS1524 BRCA1 with a corresponding increase in 53BP1 foci, activation of ATM and signaling into mutant p53. A shift to more error prone repair combined with a loss of checkpoint function leads to fragmentation of chromosomal material. The degree of disruption to DDR signalling correlated to chromosomal fragmentation and loss of clonogenicity. ATM shRNA indicated a possible rationale for the combination of AUY922 and CCRT in cells lacking ATM function.

**Conclusions:**

This study supports future clinical studies combining AUY922 and CCRT in p53 mutant HNSCC. Modulation of the DDR and chromosomal fragmentation are likely to be analytical points of interest in such trials.

## Background

Concurrent cisplatin radiotherapy (CCRT) is a standard-of-care for patients with locally advanced head and neck squamous cell carcinoma (HNSCC). Despite improving outcomes with CCRT, patients with locally-advanced HNSCC have a poor prognosis. Novel tumor-selective therapies are urgently needed, with efficacy in conjunction with existing CCRT being the most likely route to clinical development [1, 2].

HSP90 is a molecular chaperone involved in the initial folding and continued conformational maintenance of a pool of client proteins. Many of these have been identified as oncoproteins or key components in repair and cell cycle arrest following exposure to DNA damaging agents [3–5]. HSP90 inhibitors mediate sensitization through multifaceted effects and radiosensitize a broad range of genetically diverse tumor types [6–12].

HSP90 inhibition has been shown to have a significant direct impact on cell cycle and DNA repair mechanisms. HSP90 client proteins include cell cycle regulators such as CHK1, WEE1, CDK1 and CDK4 [13, 14], as well as DNA repair proteins such as ATR, FANCA, RAD51 and BRCA2 [4, 15–17]. HSP90 inhibition does not alter Ku70, Ku80 or DNA-PK total protein levels but can reduce phosphorylation of DNA-PKcs. This has been shown to be due to disruption of EGFR activity via HER2 depletion in cells lacking HER3 [17, 18]. Together with the observation that HSP90 co-localizes with γH2Ax repair foci [19], these previous findings suggest HSP90 inhibition as a promising target for radio- and chemo-sensitization studies.

AUY922 [20] is a small molecule HSP90 inhibitor (HSP90i) that is currently recruiting in Phase II trials for NSCLC and gastrointestinal stromal tumours. Previous studies reported AUY922 as a radiosensitizer and that other HSP90 inhibitors can sensitize to cisplatin alone [21–25]. Since meaningful clinical utility for HSP90i in HNSCC is most likely to be in the context of CCRT, we sought to assess the combinations of AUY922 with CCRT in p53 mutant (p53mt) HNSCC cell lines. TCGA data has shown 85% of HPV negative HNSCC harbour mutations in p53. Our goal was to thoroughly profile the impact of AUY922 on DNA damage response (DDR) signalling due to CCRT. A greater understanding of how AUY922 modulates the DDR is crucial to establishing future planning and assessment of clinical trials in p53mt HNSCC.

## Methods

### Cell culture conditions

Cal27 (CRL-2095) and FaDu (HTB-43) cells were obtained from ATCC. LICR-LON-HN5 were a kind gift from Suzanne Eccles (The Institute of Cancer Research, Sutton, London, UK). All three cell lines were HPV negative and p53 mutant. Cells were cultured in DMEM (Invitrogen, Paisley, UK) supplemented with 10% FCS, 2 mM L-glutamine and 1% penicillin/streptomycin in a humidified incubator at 37 °C with 5% CO_2_. Cells were tested for mycoplasma using the eMyco PCR kit from IntroBio (Seongnam-Si, South Korea) and authenticated by STR profiling (Bio-Synthesis Inc, Texas, US).

### Drugs and irradiation

AUY922 was kindly donated by Novartis in the form of the mesylated salt. Cisplatin was from Teva Hospitals (Castleford, UK). In western blot, confocal and FACS analysis 10 nM AUY922 or 10 μM cisplatin was used unless otherwise indicted. AUY922 was added 16 h before cisplatin or irradiation. Irradiation was carried out using an AGO 250 kV X-ray machine (AGO, Reading, UK).

### Clonogenic assay

Long-term survival in response to radiation was measured by colony formation assay. Cells were trypsinized, diluted and counted before seeding in 6-well dishes or 10 cm dishes at appropriate seeding densities. Cells were allowed to attach before addition of 5 nM AUY922 or DMSO only control for 16 h. Cells were exposed to 5 μM cisplatin for 3 h with cells subject to concurrent-cisplatin radiotherapy being irradiated immediately after cisplatin addition. After 3 h exposure to cisplatin, both cisplatin and AUY922 were replaced by drug-free medium. Colonies were fixed and stained in 5% gluteraldehyde, 0.5% crystal violet, with colonies containing more than 50 cells counted. Colony counting was performed both manually and by automated quantification using CellProfiler 2.0 (Broad Institute, MA, USA). Surviving fraction was calculated by normalization to untreated controls.

### Western blotting

Medium and cells were harvested in PBS-containing 1 mM Na_3_VO_4_ and 1 mM NaF. Cells were pelleted before lysis in 50 mM Tris.HCl pH 7.5, 150 mM NaCl, 1% NP-40, 0.5% deoxycholate and 0.1% SDS. Samples were thawed on ice, centrifuged at 14,000 rpm for 20 min at 4 °C and supernatants quantified by BCA assay from Pierce (Leicestershire, UK). 30 μg total protein lysate was separated by reducing SDS-PAGE, transferred to PVDF (GE Healthcare, Bucks, UK) and blocked with 5% non-fat dry milk in TBS. The following primary antibodies were used: rabbit anti-HSP72 from Stressgen (Exeter, UK); rabbit anti-GAPDH, rabbit anti-ATR, rabbit anti-phospho-ATR (S428), rabbit anti-CHK1, rabbit anti-phospho-CHK1 (S345), rabbit anti-RAD51, rabbit anti-ATM, rabbit anti-phospho-ATM (S1981), rabbit anti-phospho-BRCA1 (S1524), rabbit anti-phospho-p53 (S15) and rabbit anti-phospho-H2Ax (S139) were purchased from Cell Signaling (MA, USA); rabbit anti-FANCA was purchased from Bethyl Laboratories (TX, USA). Secondary antibodies used were sheep anti-mouse IgG and donkey anti-rabbit IgG HRP from GE Healthcare (Buckinghamshire, UK). Chemiluminescent detection was carried out using immobilon western substrate from Millipore (East Midlands, UK). In vivo samples were processed using a Precellys®24 homogenizer from Bertin Technologies (Montigny, France).

### Lentiviral shRNA production and infection

Short hairpin sequences were cloned into the lentiviral shRNA plasmid pHIVSiren [26]. The plasmid pHIVSiren was kindly donated by Professor Greg Towers, University College London and was derived from a parent plasmid, CSGW (Prof Adrian Thrasher, University College London). FANCA and ATM short hairpin target sequences were 5’-GTGGCATCTTCACGTACAA-3’ and 5’-GTGGCATCTTCACGTACAA-3’, respectively. Scrambled short hairpin target sequence was 5’-GTTATAGGCTCGCAAAAGG-3’. Short hairpin containing pHIVSiren was co-transfected with the packaging plasmids psPAX2, pMD2.G into HEK293T cells using lipofectamine 2000 (Life Technologies, Paisley, UK). Viral supernatants were collected and target cells infected in the presence of 1 μg per mL polybrene.

### Flow cytometry

Cells were stained for mitosis or DNA double-stranded breaks with rabbit anti-phospho-histone H3 S10 (DD2C8) AlexaFluor647 or anti-phospho-histone H2Ax S139 (20E3) AlexaFluor488 (Cell Signaling, MA, USA) using the manufacturer’s protocol. Cells were analyzed on an LSR II from BD Biosciences (Oxford, UK).

### DDR confocal image based analysis

Cells were plated in 35 mm glass-bottomed dishes (Mattek, MA, USA). Samples were fixed in 4% PFA, permeabilized in 0.2% Triton X-100 and treated with DNaseI (Roche, West Sussex, UK). Cells were blocked in 1% BSA, 2% FCS in PBS before staining with rabbit anti-phospho-H2Ax S139 (γH2Ax), rabbit anti-RAD51, rabbit anti-53BP1, anti-phospho-BRCA1 (S1524), rabbit anti-phospho-p53 (S15) or mouse anti-phospho-ATM (S1981) (Cell Signaling, MA, USA) with goat anti-rabbit Alexafluor488 or goat anti-mouse Alexfluor546 as secondary antibodies (Invitrogen, Paisley, UK). Nuclei were counterstained with DAPI. Samples were imaged using a Zeiss LSM710 inverted confocal microscope (Zeiss, Jena, Germany). Automated quantification of foci in 100-300 nuclei per experiment was carried out using CellProfiler 2.0 (Broad Institute, MA, USA). Formalin-fixed paraffin embedded (FFPE) in vivo blocks were sectioned and antigen retrieved for RAD51 (pH9 Tris-EDTA) or 53BP1 (pH6 citrate buffer). Antigen retrieved slides were blocked, stained, imaged and quantified as outlined for in vitro samples above.

### In vivo human xenograft model

Female 5-6 week-old athymic BALBc nude mice (Charles River, UK) were used with all experiments, complying with NCRI guidelines. 2x10^6^ FaDu cells were injected subcutaneously. Developing tumors were distributed into groups containing a minimum of n = 8 per group, with matching average tumor volumes. AUY922 40 mg/kg in 5% dextrose was administered in three doses by i.p. injection on days one, three and five. Fractionated radiation treatment of the tumor consisted of a total dose of 6 Gy in 2 Gy fractions on day two, four and six. Cisplatin was administered as a single dose of 5 mg/kg on day four immediately before irradiation. Tumor volume was calculated as volume = (width × length × depth)/2 and was plotted as mean tumor volume for each group.

### Statistical analysis

Statistical analysis was carried out using Graphpad prism (version 6.0f). Unpaired two-tailed student *t*-test was utilized for parametric analysis. Synergy was determined by Bliss independence analysis using the equation E_exp_ = E_x_ + E_y_ – (E_x_E_y_) [27]. E_exp_ is the expected effect if two treatments are additive with E_x_ and E_y_ corresponding to the effect of each treatment individually. ΔE = E_observed_ - E_exp_. Synergy is represented by ΔE and 95% confidence intervals (CI) from observed data all above zero; addition to values above and below zero; antagonism where all values are below zero.

## Results

### AUY922 sensitizes p53mt HNSCC to cisplatin, radiation and concurrent-cisplatin radiotherapy (CCRT)

**Fig. 1:**
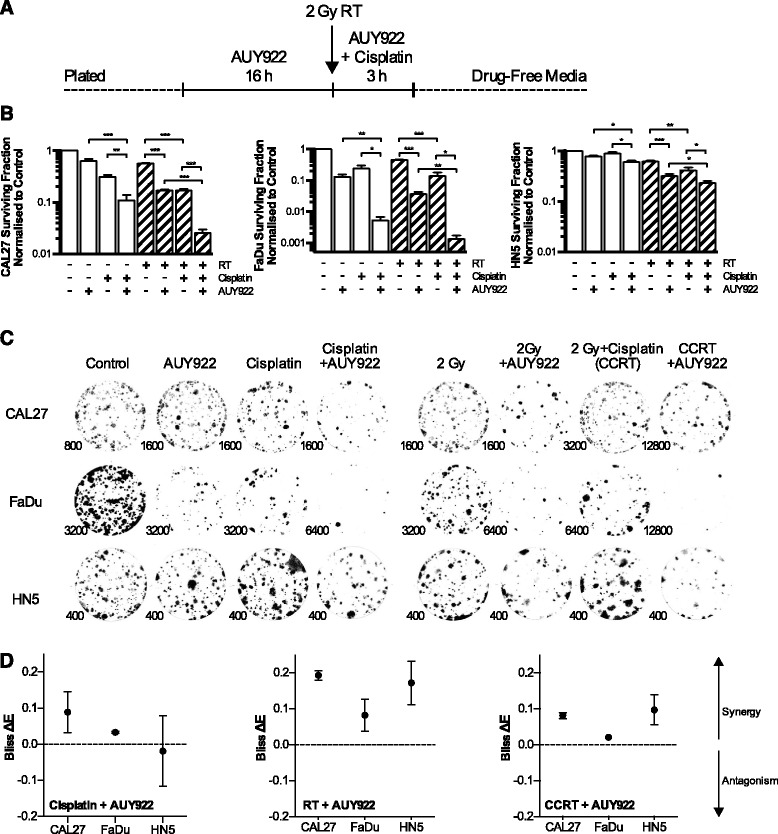
Clonogenic survival and Bliss analysis for concurrent cisplatin radiotherapy (CCRT) and AUY922. Clonogenic survival assay showing cisplatin, radiation, or CCRT sensitizing effect of HSP90i by AUY922 on p53 mutant head and neck cell lines CAL27, FaDu and HN5. **a** Clonogenic drug scheduling. 5 nM AUY922 was added 16 h before addition of 5 μM cisplatin and/or immediate irradiation. Cisplatin and AUY922 were replaced with fresh media 3 h post-radiation. **b** Surviving fractions were calculated by normalization of treated wells to the plating efficiency of untreated controls. Values ± SEM of at least 3 independent experiments. Statistical analysis by 2-tailed *t*-test; *p < 0.05, **p < 0.01, ***p < 0.001. **c** Representative images of colonies for each cell line and each condition. Numbers indicate number of cells plated per well shown. **d** Analysis of synergy for the addition of AUY922 to cisplatin, radiation or CCRT as indicated by the Bliss Independence Model plotted as ΔE values ± 95% confidence intervals. ΔE = Observed reduction in clonogenicity – Expected reduction in clonogenicity, with survival expressed as a fraction of 1. Values with confidence intervals falling above zero represent synergy, negative values antagonism

The ability of AUY922 to sensitize to cisplatin, radiation and CCRT was assessed in a panel of cell lines by clonogenic assay using the scheduling outlined (Fig. 1a). We focused our studies on p53mt since p53 pathway abnormalities exist in 85% of HPV-negative HNSCC (TCGA).

Clonogenic data are presented as surviving fractions, normalised to drug free control wells (Fig. 1b). Qualitative images of colonies for each cell line and condition are also shown with numbers indicating the number of cells plated in each well shown (Fig. 1c). Bliss independence analysis (Fig. 1d) indicated synergy for the combination of AUY922 and cisplatin, except HN5 in which the interaction was additive. Synergy for the combination of AUY922 with both radiation and CCRT was observed in all cell lines tested. Values for synergy between 0 and 0.2 are in keeping with those observed in recent radiosensitization studies for CHK1 and ATR inhibition [28, 29]. FaDu cells were substantially more sensitive to AUY922 and cisplatin monotherapies as well as AUY922 and cisplatin or CCRT combinations. HN5 cells were more resistant to monotherapies and were one to two orders of magnitude more resistant to the combination of AUY922 and CCRT compared to other cell lines.

### Inhibition of HR via RAD51 and BRCA1 corresponds to increased 53BP1 foci and signalling into mutant p53

**Fig. 2:**
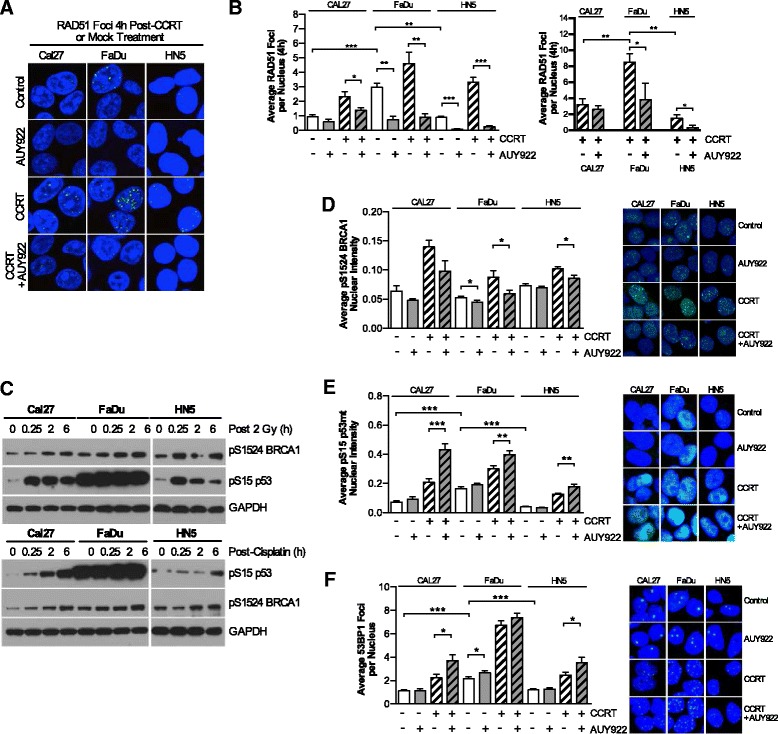
AUY922 reduces HR in response to CCRT but increases 53BP1 focal formation and mutant p53 signaling. **a** Representative images of RAD51 foci in the HNSCC cell lines CAL27, FaDu and HN5. Nuclear localization indicated by DAPI staining. AUY922 refers to 10 nM added 16 h pre-DNA damage. 2 Gy radiation plus 10 μM cisplatin for brevity is referred to as concurrent-cisplatin radiotherapy (CCRT). **b** Quantitation of the average RAD51 foci per nucleus at 4 h and for CCRT and CCRT + AUY922 conditions at 24 h also. Values shown are means ± SEM of a minimum of three independent experiments. **c** Western blot analysis of pS1524 BRCA1 and pS15 p53 signaling post irradiation or cisplatin treatment. **d**, **e**, **f** Automated image based quantification of average nuclear pS1524 BRCA1 intensity, average pS15 p53mt nuclear intensity, and average 53BP1 foci per nucleus with treatment schedule as outlined in panel **b**. Representative nuclear staining for each cell line and condition are shown. Values shown are means ± SEM from a minimum quantification of 12 fields of view across two independent experiments, except for pS1524 BRCA1 in CAL27 cells which represent 8-10 fields of view from a single experiment. Statistical analysis by 2-tailed *t*-test; *p < 0.05, **p < 0.01, ***p < 0.001

We investigated the impact of AUY922 on CCRT-induced RAD51 focus formation. CCRT induced an increase in early RAD51 focus formation which was significantly reduced by AUY922 (Fig. 2a, b). Western blots looking at DDR signalling with radiation and cisplatin alone revealed similar levels of S1524 BRCA1 in all cell lines, but diverse phosphorylation of mutant p53 S15. FaDu cells were constitutively high for pS15 p53 with levels in HN5 cells rapidly decreasing after an early radiation induced spike (Fig. 2c).

To more conclusively investigate this difference we looked at nuclear staining of pS1524 BRCA1, pS15 p53 and 53BP1 focus formation. Nuclear intensity of pS1524 BRCA1 signalling increased due to CCRT and was statistically lower due to AUY922 (Fig. 2d). Nuclear intensity of CCRT induced pS15 p53 increased in all cell lines due to the addition of AUY922 (Fig. 2e). CCRT induced 53BP1 foci increased in all cell lines due to the addition of AUY922 (Fig. 2f). Overall it was observed that FaDu cells exhibited the highest basal levels of RAD51, 53BP1 and pS15 p53 with the largest number of CCRT induced RAD51 and 53BP1 foci. HN5 cell displayed the lowest basal DDR signalling pattern and low levels of RAD51 foci persisting at 24 h as well as low levels of pS15 mutant p53. Cal27s fell in between, with this pattern correlating to the results observed in clonogenic assays (Fig. 1b).

### FANCA and RAD51 depletion by AUY922 perturbs normal RAD51 focus formation and increases ATM focus formation in response to CCRT

**Fig. 3:**
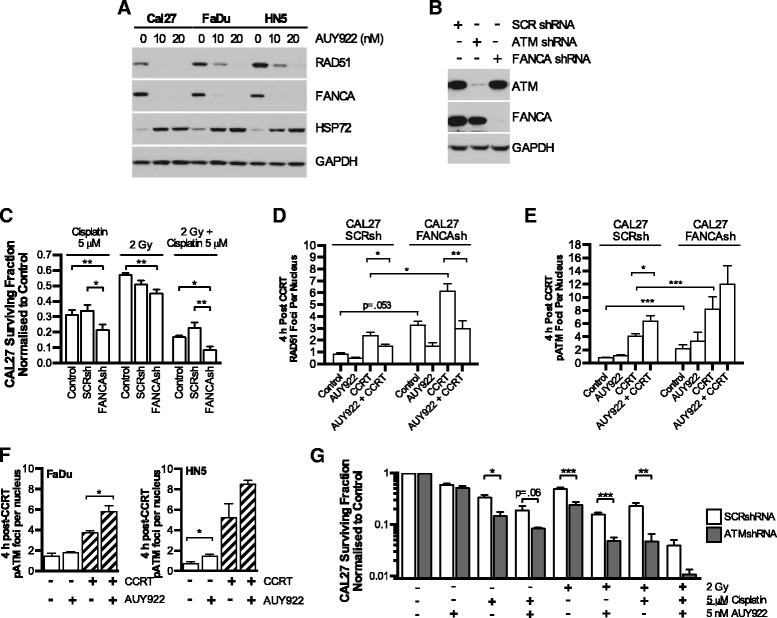
AUY922 disruption of FANCA leads to increased dependency on ATM in response to CCRT. **a** Western blot analysis of RAD51 and FANCA depletion by AUY922 in the p53mt HNSCC cell lines CAL27, FaDu and HN5. **b** Confirmation of FANCA and ATM knockdown using lentiviral shRNA by western blot in the p53 mutant HNSCC cell line CAL27 vs scrambled shRNA. **c** Clonogenic survival assay showing cisplatin; radiation; or CCRT toxicity to CAL27 cells expressing scrambled (SCR) or FANCA shRNA vs control cells with no lentiviral infection. **d**, **e** Quantification of RAD51 or S1981 phospho-ATM foci by confocal microscopy in SCRsh or ATMsh CAL27 cells. CCRT and AUY922 doses as outlined in Fig. 2. **f** S1981 phospho-ATM foci in FaDu and HN5 cells in response to CCRT and AUY922. **g** Clonogenic survival assay showing cisplatin, radiation, or CCRT sensitizing effect of HSP90i by AUY922 in scrambled of ATM shRNA expressing CAL27 cells. All values mean ± SEM of at least 3 independent experiments. Statistical analysis by 2-tailed *t*-test; *p < 0.05, **p < 0.01, ***p < 0.001

Previously reported as a HSP90 client protein, we investigated how FANCA and RAD51 depletion may impact ATM signalling (measured by autophosphorylation on S1981). AUY922 depleted both RAD51 and FANCA to similar levels in CAL27, FaDu and HN5 cells with canonical drug-on-target induction of HSP72 (Fig. 3a). Stable knockdown in CAL27 cells of FANCA and ATM by lentiviral shRNA is shown by western blot (Fig. 3b).

As expected, FANCA knockdown increased sensitivity to cisplatin and CCRT with no statistically significant difference between control and scrambled shRNA conditions (Fig. 3c). FANCA knockdown significantly increased basal RAD51 and pS1981 ATM focus formation in response to CCRT (Fig. 3d, e). CCRT-induced pS1981 ATM foci were further increased by the addition of AUY922 in all cell lines (Fig. 3e, f) coinciding with a reduction in RAD51 foci (Fig. 2b, Fig. 3d).

We then generated ATM knockdown cells to investigate the role of ATM in compensating for loss of RAD51 and FANCA due to AUY922. Knockdown of ATM in CAL27 cells resulted in increased sensitivity to cisplatin, radiation and CCRT (Fig. 3g). AUY922 was able to further sensitize to cisplatin, radiation or CCRT. Overall survival was profoundly decreased in all combinations vs scrambled.

### AUY922 abrogates ATR-CHK1 signaling and induces chromosomal fragmentation

**Fig. 4:**
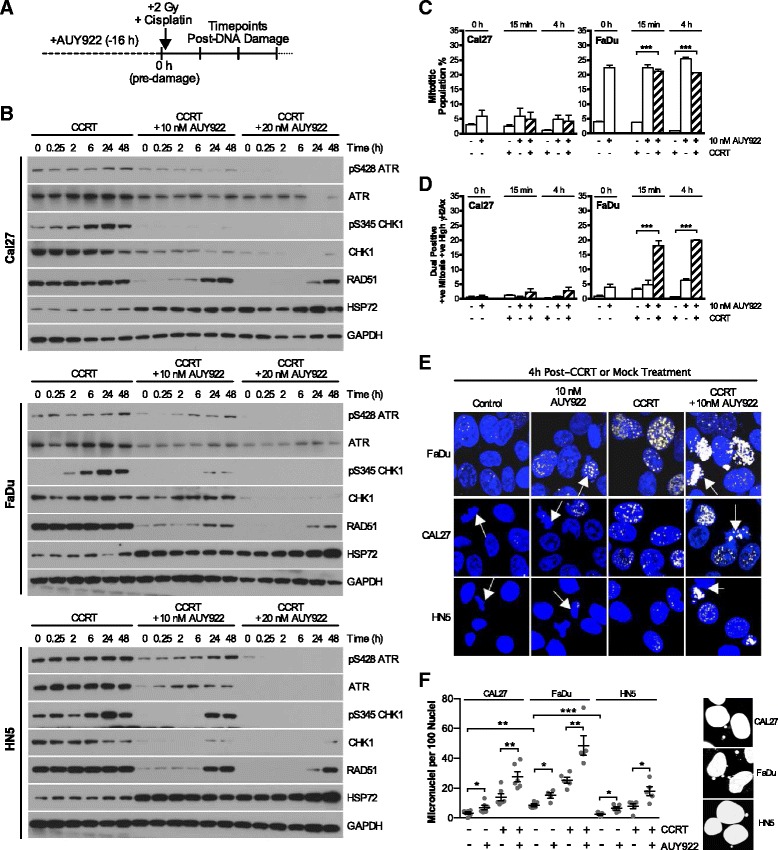
AUY922 abrogates ATR-CHK1 signaling allowing increased chromosomal fragmentation in response to CCRT. **a** Scheduling showing 0 h time point post 16 h AUY922 addition but pre-RT, cisplatin or combined CCRT addition and subsequent time point analysis post as used in panels **b**-**f. b** AUY922 disruption of ATR-CHK1 signaling in response to CCRT alongside depletion of total RAD51. **c** Mitotic accumulation as measured by FACS analysis of phospho-histone H3 positive cells. **d** Co-staining for phospho-histone H3 and γH2Ax was analyzed by FACS. Population plotted is the percentage of the total cell number positive for both high γH2Ax levels and the mitotic marker phospho-histone H3. **e** γH2Ax staining in mitotic cells was confirmed in HNSCC cell lines by confocal microscopy, DAPI as nuclear stain. Nuclei with mitotic morphology indicated by arrows. **f** Micronuclei quantification of DAPI stained confocal images at 24 h in response to CCRT and AUY922. Values are mean ± SEM of at least three independent experiments. Statistical analysis by 2-tailed *t*-test; *p < 0.05, **p < 0.01, ***p < 0.001

Following on from evidence of increased ATM foci induced by AUY922, we looked at ATR-CHK1 signaling. In all cell lines, moderate decreases in phospho-ATR, total-ATR and total-CHK1 combined to give a substantial reduction in phospho-CHK1 signaling (Fig. 4b). In studying the impact of this inhibition on mitotic entry by phospho-histone H3 staining (Fig. 4c), AUY922 was found to induce a profound increase in the mitotic population. This was much less pronounced in CAL27 cells, while HN5 cells exhibited an increase in the mitotic population from 2.9 to 8.1% due to AUY922 treatment (data not shown).

Co-staining FACS analysis of both the mitotic marker phospho-histone H3 and the double-stranded break marker γH2Ax revealed that this AUY922-induced mitotic population became highly γH2Ax positive immediately after CCRT (Fig. 4d). Confocal microscopy (Fig. 4e) further confirmed the presence of high levels of γH2Ax foci in nuclei displaying a mitotic morphology post-CCRT, as well as chromosome fragments or missegregation. We quantified the presence of micronuclei at 24 h post CCRT (Fig. 4f). AUY922 alone increased micronuclei compared to basal and significantly increased micronuclei when combined with CCRT vs CCRT alone in all cell lines.

FaDu cells showed high levels of chromosomal fragmentation both basally and in response to AUY922 plus CCRT. HN5 cells showed low basal levels and the lowest number of micronuclei in response to CCRT plus AUY922. This pattern of micronuclei formation closely aligned to that observed for RAD51, 53BP1 repair foci formation and signalling into mutant p53 (Fig. 2b, e, f). This DNA repair foci pattern and micronuclei generation correlated to the differences in sensitivity observed in clonogenic assays (Fig. 1b) with FaDus being the most sensitive and HN5s the most resistant.

### AUY922 enhances growth delay of CCRT treated FaDu HNSCC xenograft tumors

**Fig. 5:**
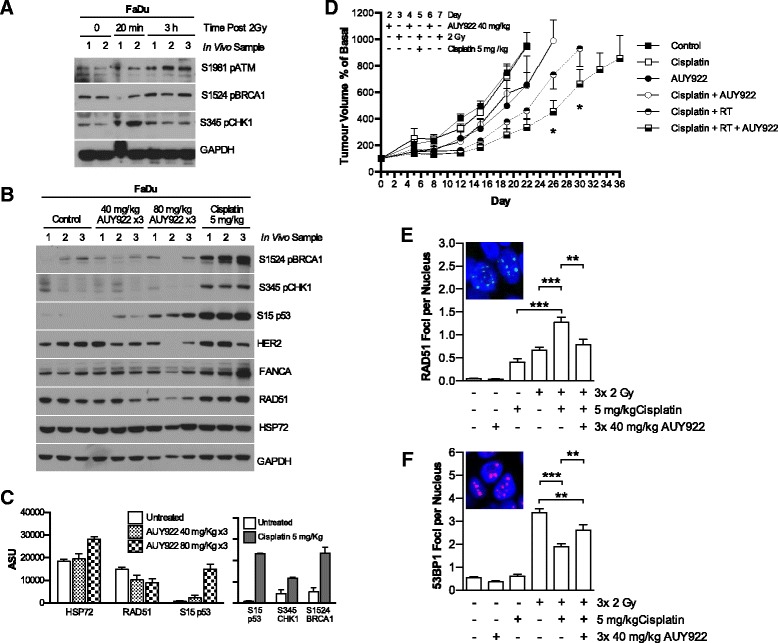
AUY922 delays tumor growth in conjunction with CCRT. **a** FaDu cells were implanted subcutaneously in BALB/c nude mice. After reaching 5-7 mm, tumors were treated with 2 Gy radiation. Tumors harvested at the times post radiation as indicated and probed for DNA damage signaling by western blot. **b** FaDu cells implanted as in A before treatment with cisplatin 5 mg/kg or three doses of AUY922 40 mg/kg on alternate days. Tumors treated with AUY922 were collected 16 h post final injection, cisplatin 24 h post injection. Western blot analysis performed for DNA damage signaling in response to cisplatin or reduction in HSP90 client proteins by AUY922. **c** Densitometry of changes due to HSP90 inhibition and response to cisplatin as shown in panel **b**, expressed as arbitrary scanning units adjusted for changes in GAPDH levels. **d** FaDu cells implanted as in A. Tumors were distributed into the following treatment groups with matching average tumor volumes; control; Cisplatin 5 mg/kg; AUY922 40 mg/kg × 3; cisplatin 5 mg/kg plus AUY922 40 mg/kg × 3; cisplatin 5 mg/kg plus three fractions of 2Gy; cisplatin 5 mg/kg plus three fractions of 2 Gy plus AUY922 40 mg/kg × 3. Exact scheduling as outlined in methods. Tumor volume expressed as percentage increase over basal volume at start of treatment. **e, f** FFPE blocks were sectioned and stained for RAD51 and 53BP1 foci. Automated quantification shown represents a minimum of 36 randomly distributed fields of view for RAD51 across 2 tumor blocks, 16-24 fields of view across for 53BP1 foci. Values ± SEM, statistical analysis by 2-tailed *t*-test; *p < 0.05, **p < 0.01, ***p < 0.001

DNA damage signaling basally and in response to radiation was assessed in FaDus in vivo (Fig. 5a). Cisplatin treatment of FaDu xenografts was confirmed to increase DNA damage signaling 24 h after 5 mg/kg cisplatin injection (Fig. 5b, c). Depletion of HSP90 client proteins by AUY922 in FaDu xenografts was assessed at both 40 mg/kg and 80 mg/kg with three IP injections on days 1, 3 and 5. Tumor lysates were collected 24 h after final injection. HER2 as a known and highly sensitive HSP90 client protein was also assessed. Reductions in RAD51 and HER2 were observed at 40 mg/kg with increased S15 phospho-p53 signaling also detected (Fig. 5b, c).

The lowest dose of 40 mg/kg AUY922 was selected to look for sensitization effects in tumor volume experiments (Fig. 5d). Mice were treated when tumors reached 5-7 mm in width with groups composed of mice with equal average tumor volume. AUY922 40 mg/kg was administered by IP injection on days 1, 3 and 5. Radiation was delivered in 3 × 2 Gy fractions on days 2, 4 and 6 with 5 mg/kg cisplatin administered by IP injection before irradiation on day 4. CCRT combined with AUY922 was tolerable with average weight loss of <10% (data not shown). The addition of AUY922 successfully delayed time to reach 800 mm^3^ from 27 days for CCRT only to 34 days from CCRT plus AUY922.

To assess DDR signalling at the level of repair foci formation in vivo, staining for RAD51 and 53BP1 was carried out on sections from FFPE tumour blocks which were all fixed 16 h after the final radiation fraction. Cisplatin and radiation combined to increase RAD51 foci in vivo*,* with AUY922 at the 40 mg/kg dose used in therapy experiments able to reduce RAD51 focus formation (Fig 5e). 53BP1 focus formation as a result of radiation decreased due to the addition of cisplatin. AUY922 addition to CCRT in increased the number of 53BP1 foci detected. These findings are in line with those shown in vitro (Fig. 2b, f).

## Discussion

The standard-of-care for locally advanced HNSCC is CCRT, yet almost 50% of patients do not survive past 5 years [30]. The anti-EGFR-targeting monoclonal antibody cetuximab is the only targeted therapy approved for HNSCC treatment. However, the RTOG 0522 phase III study showed there was no benefit from adding cetuximab to cisplatin-based CCRT [31]. Cetuximab illustrates that success in clinical trials is likely to be measured by the capability to improve survival as an addition to CCRT rather than with radiation alone.

Our goal in this study was to iterate on the already established ability of HSP90 inhibition to radiosensitize. We set out to determine if HSP90 inhibition in combination with CCRT was likely to offer a significant stepwise improvement or if the addition of cisplatin had the potential to interfere with radiation sensitization by AUY922. The addition of AUY922 to cisplatin, radiation and CCRT combinations was shown to be synergistic across a panel of p53mt. AUY922 and was capable of enhancing the efficacy of CCRT in vivo.

Sensitization to CCRT by HSP90i has previously been published in both NSCLC [21] and bladder cancer [25]. Wang et al. examined the ability of HSP90i by ganetespib to sensitize a panel of NSCLC KRAS mt p53 wt and KRAS wt p53 mt/null cell lines [21]. Ganetespib radiosensitized all cell lines but they showed HSP90i produced variable results both in vitro and in vivo to carboplatin-paclitaxel and concomitant carboplatin-paclitaxel and radiation. The use of paclitaxel-carboplatin rather than carboplatin alone complicates interpretation of these results relative to our study. We see broad sensitization to CCRT while they see cases of antagonism by HSP90i. This could be cell line specific or related to paclitaxel. Yoshida et al. assessed cisplatin and radiation in bladder cancer cell lines showing sensitization by 17-DMAG to radiation and CCRT [25]. While a number of studies have looking at HSP90i sensitization to radiation or cisplatin individually in head and neck [12, 24, 32], none extensively address the ability of HSP90i to sensitize p53mt HNSCC to concurrent-cisplatin radiotherapy.

We concentrated on investigating the ability of AUY922 to disrupt HR induced by CCRT and other DDR signalling pathways by extensive confocal image based analysis. RAD51, BRCA1 and BRCA2 have previously been identified as HSP90 client proteins, with depletion of RAD51 and RAD52 occurring upon loss or inhibition of HSP90 isoforms in budding yeast [17, 23, 33]. Previous mechanistic studies on HSP90i have not focused extensively on DDR signalling. In the HSP90i and platinum-radiotherapy combinations mentioned above, 53BP1 foci alone were analysed but only for ganetespib and radiation [21]. For HSP90i and CCRT in bladder cancer, mechanistic studies focused on HER2 and AKT signalling with no investigation of the impact of HSP90i on DDR signalling [25]. Likewise studies into sensitization to radiation or cisplatin alone often focused on cell cycle, growth and apoptotic signalling pathways [22, 24, 32, 34–36]. Choi et al. identified HSP90i by bioinformatics as a means to convert HR proficient to HR deficient tumours [23] but DDR analysis was restricted to γH2Ax and RAD51 foci formation as has been the case in other studies [17, 22, 35].

In this study we comprehensively profiled HSP90i modulation of the DDR to CCRT. Reduction in HR by HSP90i occurs due to decreased RAD51 focus formation and nuclear pS1524 BRCA1. This corresponds to HSP90i induced increases in 53BP1 foci. This may be in part a separate inhibitory event on the resolution of 53BP1 repair sites or a switch from HR to NHEJ. 53BP1 has been identified to antagonise DSB end resection promoting NHEJ over HR. It has been proposed that 53BP1 is displaced in S-phase in a BRCA1 dependent manner. The role of BRCA1 in promoting HR over NHEJ through 53BP1 has been recently reviewed [37, 38]. This suggests HSP90i via a reduction in nuclear BRCA1 signalling may also shift HR to more error prone NHEJ repair rather than a delay in existing 53BP1 foci resolution alone. Modulation of DDR at the repair foci level in vivo has also been demonstrated for the first time in FFPE blocks. This may be a beneficial for analysis of future clinical trials where FFPE biopsies are more routinely used for analysis.

HSP90i increased CCRT induced nuclear pS15 p53mt levels. The role this increased p53mt signalling may play is not known. The early HSP90 inhibitor 17-AAG has been shown to stabilise wild type p53 in head and neck cell lines through a reduction in MDMX increasing apoptosis in response to cisplatin [24]. Parallel studies were not performed on mutant p53.

**Fig. 6:**
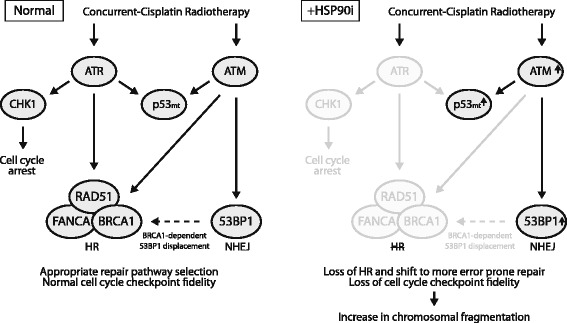
Overview of HSP90i modulation of CCRT induced DDR signaling in p53mt HNSCC. Simplified schematic showing specific changes to DDR proteins due to AUY922 as observed in in Figs. 2, 3, 4 and 5 and in the context of the literature as outlined in the discussion

In exploring the role the HR component FANCA may play in HSP90 chemosensitization, we discovered a profound increase in ATM foci in response to AUY922. FANCA is part of the Fanconi Anemia core complex that ubiquitinates FANCD2 at interstrand crosslink sites, leading to crosslink unhooking, lesion bypass and downstream completion of repair by RAD51-mediated HR [39]. FANCA depletion alone by shRNA revealed an increase in RAD51 alongside increased ATM focus formation. It is not known if FANCA loss results in a numerical increase in the incidence of damage requiring RAD51 and ATM focus formation or simply prevents the timely resolution of existing cisplatin adducts leading to accumulation. The exact cause of this increased ATM signal due to AUY922 is hard to pinpoint. ATM is autophosphorylated on Ser1981 [40]. FANCA mutation and ATR loss have both been shown to increase phosphorylation of S1981 ATM and S15 p53 [41, 42] with ATM known to phosphorylate S15 of p53 in response to DNA damage [43]. This suggests decreased levels of ATR and FANCA by HSP90i lead to compensatory signalling via ATM and p53 in response to CCRT. An illustration of the hypothesised changes in CCRT induced DDR signalling triggered by HSP90i and downstream consequences is summarised in Fig. 6.

Decreased RAD51, FANCA and ATR function by HSP90 inhibition may lead to increased dependence on ATM for repair. Cells subject to ATM knockdown by shRNA were substantially more sensitive to cisplatin, RT and CCRT alone and in combination with HSP90i. Loss of ATM has been shown to occur in head and neck due to loss of the distal region of chromosome 11q [44]. Much discussion has occurred around the potential to target ATM loss as a synthetic lethal strategy [45]. ATR inhibition alone is being investigated as a radiosensitizer with some studies showing ATR inhibition leading to increased dependency on ATM [46, 47].

The ultimate consequence of a shift to more error prone repair and loss of S-phase and G2/M checkpoint fidelity was missegregation of chromosomal material and micronucleus formation. We observed that the most sensitive cell line in clonogenic assays (FaDu) displayed the highest levels of DDR signalling due to CCRT and the highest levels of chromosomal fragmentation with the addition of HSP90i. The least sensitive cell line in clonogenic assays (HN5) displayed the lowest levels of both DDR signalling and chromosomal fragmentation. Micronuclei deficient in nuclear import, prone to rupturing and incomplete replication [48, 49] are putatively the major toxic event induced by AUY922 inhibition in combination with CCRT.

## Conclusions

In summary, this study demonstrated inhibition of HSP90 by AUY922 had a synergistic interaction with CCRT in a panel of p53 mutant cell lines. HSP90i leads to significant alterations to the DDR induced by CCRT. This comprises inhibition of HR, a shift to more error prone repair and loss of checkpoint function leading to fragmentation of chromosomal material. Additionally, these results indicate there may be a rationale for the combination of AUY922 and CCRT in cells lacking ATM function. In conclusion, these data show that HSP90 inhibition can improve upon CCRT standard-of-care and support further preclinical and clinical studies in HNSCC.

## Abbreviations

CCRT: Concurrent-cisplatin radiation
CI: Confidence interval
DDR: DNA damage response
HNSCC: Head and neck squamous cell carcinoma
HR: Homologous recombination
HSP: Heat shock protein
p53mt: p53 mutant
